# Factors associated with loneliness in middle-aged and older patients with breast cancer

**DOI:** 10.1016/j.apjon.2024.100444

**Published:** 2024-03-18

**Authors:** Leni Merdawati, Hui-Chen Lin, Ya-Ching Wang, Kuan-Chia Lin, Hui-Chuan Huang

**Affiliations:** a School of Nursing, College of Nursing, Taipei Medical University, Taiwan; b Faculty of Nursing, Universitas Andalas, West Sumatera, Indonesia; c School of Nursing, College of Medicine, National Taiwan University, Taipei, Taiwan; d Cheng Hsin General Hospital, Taipei, Taiwan; e Taiwan and Community Medicine Research Center, National Yang Ming Chiao Tung University, Taipei, Taiwan

**Keywords:** Breast cancer, Frailty, Loneliness, Social support

## Abstract

**Objective:**

Loneliness is associated with adverse mental and physical health conditions and increased mortality. In this study, we identified significant factors associated with loneliness in middle-aged and older patients with breast cancer (BC).

**Methods:**

For this cross-sectional study, we enrolled 200 patients (aged from 20 to 60 years) with BC from two hospitals in Indonesia through convenience sampling. Demographic characteristics, distress symptoms (Symptom Distress Scale), social support (Multidimensional Scale of Perceived Social Support), frailty (Groningen Frailty Indicator), and loneliness (UCLA Loneliness Scale, version 3) were measured. Multivariate logistic regression was performed to identify significant factors associated with loneliness in our cohort.

**Results:**

Loneliness risk was negatively correlated with social support but positively correlated with unemployment and frailty. Thus, the patients received a high level of social support (odds ratio [OR]: 0.96; 95% confidence interval [CI]: 0.92–0.99) and had a low risk of severe loneliness. By contrast, patients who were unemployed (OR: 4.00; 95% CI: 1.65–9.66) and those who had frailty (OR: 5.79; 95% CI: 2.50–13.42) had an elevated risk of severe loneliness.

**Conclusions:**

Unemployment, social support, and frailty may significantly influence the risk of loneliness in patients with BC. Early and regular assessments of loneliness should be integrated in the care of these patients. Suitable strategies aimed at increasing social support and mitigating frailty may benefit middle-aged and older patients with BC, particularly unemployed patients, by reducing their risk of loneliness.

## Introduction

Loneliness is a negative emotion resulting from a lack of social relationships or a mismatch between social relationships and individual expectations.^1^ Loneliness due to social isolation is a common health problem in older populations.^2^^,^^3^ Social isolation because of cancer-related changes in the physiological and mental health of patients with cancer and dissatisfaction with social relationships may contribute to loneliness.^4^^,^^5^ A high prevalence of loneliness (27.0% to 44.7%) has been reported in patients with cancer.^6^ Notably, patients with cancer who experience loneliness exhibit a pronounced inflammatory response to acute stress; this compromises their quality of life and increases their risk of mortality.^7^^,^^8^ A meta-analysis suggested that loneliness, a negative emotion, is a predisposing factor for depression.^9^ Loneliness has been identified as a determinant of depression in patients with breast cancer (BC).^10^ Thus, assessing and managing loneliness in patients with cancer are imperative to prevent depression and improve clinical outcomes.

Approximately 50.0% of all patients with BC experience loneliness after their cancer diagnosis.^11^ A study reported an increasing trend in loneliness in the first year after cancer diagnosis, with 22.0% to 35.0% of women aged ≥ 50 years reporting loneliness.^12^ Patients with BC often experience body image disturbances secondary to various treatments (e.g., partial or complete breast removal), severe scarring, and breast alterations, which may limit their participation in social activities.^13^^,^^14^ Moreover, various treatment side effects, including hair loss, skin changes, nausea, and fatigue, reduce their motivation to engage in these activities.^15^ In developing countries such as Indonesia, patients with BC may experience stigmatization and social discrimination due to their diagnosis.^16^ Stigmatization may subject patients with BC to perceived embarrassment, unkindness, and blame, all of which are associated with loneliness.^17^^,^^18^ This necessitates early assessment and management of loneliness in patients with BC, particularly those residing in developing countries such as Indonesia.

Loneliness is a public health problem that compromises individuals' physical and mental health.^2^ Unemployment, cancer symptoms (e.g., pain and fatigue), cognitive decline, and low social support increase the risk of loneliness in patients with cancer.^6^^,^^19^ Moreover, frailty has been associated with a risk of loneliness in older cancer survivors.^20^^,^^21^ However, few studies have investigated the correlations of these factors with a risk of loneliness in middle-aged and older patients with BC. Educational programs and psychological interventions, such as coping skill training, stress management, psychological support, and supportive-expressive therapy, have proven effective in mitigating loneliness in patients with cancer or BC.^22^^,^^23^ Addressing loneliness aligns with the principle of patient-centered care, which emphasizes prioritizing patients’ overall well-being. Thus, factors influencing loneliness in middle-aged and older patients with BC should be identified.

According to the theory of unpleasant symptoms, three groups of antecedents—physiological factors (e.g., disease characteristics and symptoms of distress), psychological factors (e.g., mood and cognition), and situational factors (e.g., social support and socioeconomic status)—are associated with unpleasant symptoms.^24^ Because loneliness is a major unpleasant symptom and a key risk factor for depression, studies must be conducted to identify the determinants of loneliness in patients with BC. Thus, this study was conducted to identify factors influencing loneliness in middle-aged and older patients with BC. For this, we explored various physiological factors (e.g., age, gender, cancer stage, cancer symptom, and frailty) and situational factors (e.g., social support). Identification of factors significantly associated with loneliness may guide the development of appropriate strategies for mitigating loneliness in middle-aged and older patients with BC.

## Methods

### Study design, setting, and sample

This cross-sectional study included patients who have visited two hospitals in West Sumatera, Indonesia, between August and October 2022. The patients were enrolled through convenience sampling. The inclusion criteria were as follows: being aged 20–60 years, receiving a BC diagnosis at least 6 months before the initiation of this study, being able to communicate in the Indonesian language, and exhibiting willingness to provide written informed consent. We excluded patients who had received a diagnosis of cognitive impairment, psychiatric disorder (e.g., schizophrenia and psychosis), psychological disorder, or depression before the diagnosis of BC. This study was conducted and is reported in accordance with the Strengthening of the Reporting of Observational Studies in Epidemiology statement guidelines,^25^ which is a checklist of items that should be addressed in articles reporting cohort, case–control, and cross-sectional studies. For a cross-sectional study, this statement recommends 22 items related to the title, abstract, introduction, methods, results, and discussion for improving the quality of reporting.

The sample size for this study was calculated considering a two-tailed α of 0.05, a power of 0.80, and an effect size of 0.30–0.48, indicating weak to moderately strong correlations of loneliness with social support and age.^26^^,^^27^ The calculations indicated that 200 patients were required for this study.

### Measurements

#### Demographic and disease characteristic questionnaire

We acquired data on the patients’ demographic characteristics, such as age, marital status, education level, the number of children, and employment status. In addition, information on the following disease characteristics was collected: cancer stage and duration.

#### UCLA loneliness scale

Loneliness is defined as a specific and unpleasant feeling of being isolated due to a lack of social relationships or an incompatibility between social relationships and individual expectations.^1^ The Indonesian version (18 items) of the UCLA Loneliness Scale was used to assess the patients’ subjective loneliness experiences. Each item is rated on a 4-point Likert scale: 1 (*never*), 2 (*rarely*), 3 (*sometimes*), and 4 (*always*). Higher scores indicate a higher severity of loneliness.^28^ The patients were stratified by the total score into the following two groups: the low-loneliness group (< 36 points), comprising patients who had never or rarely experienced loneliness, and the high-loneliness group (≥ 36 points), comprising patients who had occasionally or always experienced loneliness.^1^ In a previous study, the Cronbach α value of the Indonesian version of the UCLA Loneliness Scale was 0.87.^28^ In our study, this value was 0.92, indicating acceptable internal reliability.

#### Symptom distress scale

Symptom distress refers to the level of discomfort patients with cancer experience from specific symptoms. This parameter was measured using the 13-item Symptom Distress Scale.^29^^,^^30^ Patients are asked to rate their distress related to the following symptoms: nausea frequency, nausea intensity, appetite, insomnia, pain frequency, pain intensity, fatigue, bowel pattern, concentration, appearance, breathing, worry or fear about the future, and coughing. Each symptom is rated on a 5-point scale, with end points ranging from 1 (*no distress*) to 5 (*severe distress*). The total score ranges from 13 to 65. A higher score indicates a higher level of symptom distress.^30^

#### Multidimensional scale of perceived social support

Social support is defined as the perceived or actual instrumental and expressive support provided by the community, social networks, and confiding partners.^31^ This parameter was measured using the Indonesian version of the Multidimensional Scale of Perceived Social Support (MPSS). The MPSS is a 12-item scale that evaluates self-perceived social support from three sources: friends (four items), family (four items), and significant others (four items). Each item is rated on a 7-point Likert-type scale, with end point ranging from 1 (*very strongly disagree*) to 7 (*very strongly agree*). The total score ranges from 12 to 84. A higher score indicates a higher level of perceived social support. A previous study indicated that the Indonesian version of the MPSS had acceptable reliability with a Cronbach's α of 0.85.^32^ The Cronbach's α of the Indonesian version of the MPSS in this study was 0.92.

#### Groningen frailty indicator

Frailty is defined as the loss of physical, cognitive, social, or psychological resources and functions, which renders an individual less capable of handling stressors.^33^ This parameter was measured using the Groningen Frailty Indicator (GFI). The GFI assesses 15 items across three domains: daily activities (four items), psychosocial functioning (five items), and health problems (six items). The score considers eight items requiring a categorical response (yes = 0, no = 1), six items requiring an ordinal response with categories 0 (*no*) and 1 (*yes or sometimes*), and one item requiring a response on a 10-point scale for physical fitness (end points ranging from 1 [*very bad*] to 10 [*very good*]). The total score ranges from 0 to 15. A score of ≥ 4 indicates frailty.^34^^,^^35^ In our study, the Cronbach α value of the Indonesian version of the GFI was 0.70, indicating acceptable internal reliability.

### Data collection

All participants were recruited from the surgical outpatient departments of two hospitals in Indonesia. They received a comprehensive explanation regarding the study objectives and their right to confidentiality. All patients provided informed consent. The aforementioned questionnaires were completed during a 20-min face-to-face interview, which was conducted by the principal researcher (Leni Merdawati) in a room in the outpatient department. After the interview, the questionnaires were reviewed to ensure completeness and avoid any missing data.

### Data analysis

Data were analyzed using SPSS (version 22.0; IBM Corporation, Armonk, NY, USA). Descriptive statistics are presented in terms of the frequency and percentage values for categorical variables and the mean and standard deviation values for continuous variables. Loneliness was defined as a categorical variable with low (≤ 36 points) and high loneliness (> 36 points) groups for data analysis to identify significant factors related to the high-loneliness group. The associations of loneliness with demographic and disease characteristics, symptom distress, social support, and frailty were investigated using the *χ^2^* or Mann–Whitney *U* test. Logistic regression (LR) was performed to identify factors influencing loneliness. Initially, variables with a *P* value of ≤ 0.05 in the chi-square test or Mann–Whitney *U* test were included in univariate LR models. Subsequently, a multivariate LR model was used to identify factors significantly influencing loneliness in patients with BC. This model included all significant variables from the univariate analysis (*P* < 0.050). All tests were two-tailed, and a *P* value of < 0.050 indicated statistical significance.

### Ethical considerations

This study was approved by the institutional review boards of Taipei Medical University (IRB No. N202206028) and Health Research Ethics Committee RSUP Dr. M. Djamil Padang Hospital (IRB No. LB.02.02/5.7/129/2022). Written informed consent was obtained from all participants. Data were anonymized, securely stored, and accessible only to the research team. Interviews were conducted in private settings, and participants were assured of the confidentiality of their responses. Study findings and data presentations do not contain any identifiable information to maintain patient confidentiality.

## Results

### Participant demographics and measurement outcomes

This study included 200 patients with BC (mean age: 46.72 ± 7.29 years). Of the patients, approximately 59.5% were aged < 50 years, 82.0% were married, 19.5% were educated till elementary or junior high school, and 55.0% were unemployed. The mean number of children was 2.39 ± 1.26. Regarding disease characteristics, 51.5% of the patients had stage III or IV cancer. The average disease duration was 34.45 ± 24.83 months.

Regarding loneliness, 41.5% of the patients experienced a high severity of loneliness (score: 37–63 points; sometime or persistent loneliness). The patients’ mean scores for symptom distress and social support were 22.67 ± 5.45 (mild symptom distress) and 62.22 ± 14.33 (moderate social support), respectively. Of the patients, 53.5% had frailty (Table 1).

**Table 1:** Demography and disease characteristics, symptom distress, social support, frailty, and loneliness (*N* = 200).

Variables	Mean ± SD	Range	*n* (%)
Age (years)	46.72 ± 7.29	27–60	
< 50			119 (59.5)
≥ 50			81 (40.5)
Marital status			
Married			164 (82.0)
Others^a^			36 (18.0)
Educational level^b^			
High			161 (80.5)
Low			39 (19.5)
Number of children	2.39 ± 1.26		
Employment status			
Employed			90 (45.0)
Unemployed			110 (55.0)
Cancer stages			
I and II			97 (48.5)
III and IV			103 (51.5)
Disease duration	34.45 ± 24.83	7–132	
Symptom distress	22.67 ± 5.45	13–41	
Social support	62.22 ± 14.33	12–84	
Frailty status	3.59 ± 2.51	0–10	
No frailty (< 4)			93 (46.5)
Frailty (≥ 4)			107 (53.5)
Loneliness	34.62 ± 12.08	18–63	
Low loneliness (≤ 36)			117 (58.5)
High loneliness (> 36)			83 (41.5)

M ± SD: mean ± standard deviation.

a Others of marital status: widowed, divorced, or single.

b Education level, low: elementary school and junior high school; high: senior high school, bachelor's degree, master's degree, and doctoral degree.

### Associations of loneliness with various factors

As shown in Table 2, loneliness was significantly associated with age (*P* = 0.049), marital status (*P* = 0.024), employment status (*P* < 0.001), symptom distress (*P* < 0.001), social support (*P* < 0.001), and frailty (*P* < 0.001). The high-loneliness group was younger than the low-loneliness group (45.53 vs. 47.63 years, respectively). The proportion of married patients was lower in the high-loneliness group than in the low-loneliness group (74.7% vs. 87.2%, respectively). The rate of unemployment was higher in the high-loneliness group than in the low-loneliness group (86.7% vs. 32.5%, respectively). Furthermore, compared with the low-loneliness group, the high-loneliness group had an elevated mean score for symptom distress (21.34 vs. 24.64, respectively), a low mean score for social support (69.02 vs. 52.62, respectively), and a high prevalence of frailty (30.8% vs. 85.5%, respectively).

**Table 2:** The association between demography characteristics, disease characteristics, symptom distress, social support, frailty, and loneliness (*N* = 200).

Variables	Low loneliness	High loneliness	Mann–Whitney/*χ*^2^	*P*
	(*n* = 117)	(*n* = 83)		
	Mean ± SD/*n* (%)	Mean ± SD/*n* (%)		
Age, years	47.63 ± 7.33	45.53 ± 7.12	−1.97	0.049
Marital status			5.13	0.024
Married	102 (87.2)	62 (74.7)		
Others^a^	15 (12.8)	21 (25.3)		
Educational level^b^			1.91	0.167
High	98 (83.8)	63 (75.9)		
Low	19 (16.2)	20 (24.1)		
Number of children	2.34 ± 1.24	2.54 ± 1.33	−0.47	0.639
Employment status			57.78	< 0.001
Employed	79 (67.5)	11 (13.3)		
Unemployed	38 (32.5)	72 (86.7)		
Cancer stages			3.23	0.072
I and II	63 (53.8)	34 (41.0)		
III and IV	54 (46.2)	49 (59.0)		
Disease duration (months)	34.93 ± 24.94	33.83 ± 24.82	−0.18	0.856
Symptom distress	21.34 ± 5.52	24.64 ± 4.73	−4.76	< 0.001
Perceived social support	69.02 ± 12.73	52.62 ± 10.63	−8.54	< 0.001
Frailty status			58.55	< 0.001
No frailty	81 (69.2)	12 (14.5)		
Frailty	36 (30.8)	71 (85.5)		

a Others of marital status: widowed, divorced, or single.

b Education level, low: elementary school and junior high school; high: senior high school, bachelor's degree, master's degree, and doctoral degree.

### Determinants of loneliness

The univariate analysis indicated age, marital status, employment status, symptom distress, social support, and frailty as significant factors associated with a high severity of loneliness (*P* < 0.001). However, the multivariate analysis revealed that only unemployment, frailty, and social support were significantly associated with a high severity of loneliness. As shown in Table 3, loneliness was positively correlated with unemployment (odds ratio [OR]: 3.58; 95% confidence interval [CI]: 1.44–8.86) and frailty (OR: 5.64; 95% CI: 2.43–13.07) but negatively correlated with social support (OR: 0.94; 95% CI: 0.91–0.97).

**Table 3:** Determinants of loneliness in patients with breast cancer: logistics regression model.

Variables	Univariate LR	*P* value	Multivariate LR	*P* value
	Crude OR (95% CI)		Adjust OR (95% CI)	
Age	0.96 (0.92, 0.99)	0.045	0.97 (0.92, 1.02)	0.259
Marital status				
Married	Ref		Ref	
Others^a^	2.30 (1.11, 4.79)	0.026	2.29 (0.82, 6.39)	0.113
Employment status				
Employed	Ref		Ref	
Unemployed	13.61 (6.47, 28.61)	< 0.001	3.58 (1.44, 8.86)	0.006
Symptom distress	1.13 (1.06, 1.19)	< 0.001	1.04 (0.96, 1.13)	0.279
Perceived social support	0.89 (0.87, 0.93)	< 0.001	0.94 (0.91, 0.97)	< 0.001
Frail status				
No frailty	Ref		Ref	
Frailty	13.31 (6.44, 27.54)	< 0.001	5.64 (2.43, 13.07)	< 0.001

CI, Confident interval; LR, Logistic regression.

a Others of marital status: widowed, divorced or single.

## Discussion

Our findings indicated unemployment, social support, and frailty as significant factors influencing the risk of loneliness in middle-aged and older patients with BC. As a pivotal public health concern, loneliness affects health-care expenditure and all-cause mortality.^36^^,^^37^ Patients with BC who experience loneliness have an increased risk of depression.^10^ Thus, improving social support, mitigating frailty, and facilitating employment can serve as practical strategies for preventing or managing loneliness, thereby reducing the risk of depression, in this population.

We observed that among patients aged 27–60 years, those who were unemployed had a higher severity of loneliness than those who were employed. A recent systematic review reported a 40.0% increase in the risk of loneliness in unemployed individuals.^38^ Furthermore, unemployment is strongly associated with loneliness in patients with lung cancer.^19^ Unemployment can lead to isolation, a reduced sense of belonging, low motivation, and poor performance or productivity, thereby intensifying the feeling of loneliness.^38^^,^^39^ Moreover, unemployed individuals tend to interact with others less frequently and feel a lower sense of belonging than do employed individuals. Thus, unemployed middle-aged patients with BC may be susceptible to severe loneliness. Thus, targeted interventions for reducing loneliness should be prioritized for these individuals.

In this study, a higher level of social support was associated with a lower risk of severe loneliness. Social support was defined as a patient's perceived instrumental and emotional support from their family members, friends, and significant others.^31^ Cancer survivors may have various concerns and experience uncertainties related to disease prognosis and posttreatment life.^40^ Patients with BC who received emotional support during chemotherapy experienced reduced uncertainty, which enabled them to adopt a proactive and self-controlled approach.^41^ Adequate social support indicates sufficient interpersonal interactions for expressing feelings and the presence of someone who provides love and affection.^42^ Thus, early assessment and enhancement, if needed, of social support are crucial for preventing loneliness in middle-aged and older patients with BC.

Evidence suggests that older cancer survivors with frailty are more likely to report loneliness than are those without frailty.^43^ Among our patients aged 27–60 years, those with frailty had a higher risk of loneliness than did those without frailty. The correlation between frailty and loneliness can be attributable to difficulties in performing physical activity and reduced social participation. Adults with cancer often exhibit a reduction in physical activity level due to treatment side effects, such as fatigue and impaired muscle function.^44^^,^^45^ Moreover, chemotherapy-induced body image concerns, such as hair loss, eyebrow loss, pigmentation-related changes, edema, and other problems, can lead to a fear of judgment and consequent avoidance of social interactions, particularly in BC survivors.^46^^,^^47^ Such physical and psychological barriers may reduce patients’ participation in social activity, thereby increasing the risk of loneliness. Thus, early and regular assessments of frailty are essential in the care of middle-aged and older patients with BC. Implementing appropriate strategies for mitigating frailty can help prevent or manage loneliness in these patients.

In Indonesia, women with BC often experience fear and shame; these feelings are associated with stigmatization and isolation within their communities.^16^^,^^48^ The stigma surrounding women with BC includes perceptions of them as having a short life span, being “sufferers,” bringing disgrace to their family members, representing bad luck, and being “imperfect” women.^16^ This stigma, which is rooted in cultural beliefs and societal norms, deters patients from seeking timely BC screening, medical assistance, and necessary counseling and support at an early stage. Stigmatization intensifies the feelings of isolation and loneliness because patients may fear judgment or misunderstanding from those around them. A study indicated that BC-related stigma is prevalent across South Asian countries, such as Indonesia, Pakistan, India, Bangladesh, and Sri Lanka, where it fosters secrecy and creates substantial barriers to accessing health care.^49^ Thus, support groups and consultation services offering emotional and informational support should be established for preventing or managing loneliness, particularly in patients with BC. Health-care professionals in South Asia require training in cultural competence to effectively satisfy the health-care needs (e.g., loneliness management) of patients with BC. Our findings indicate the need for strategies aimed at increasing BC awareness, educating communities to eliminate detrimental stigmas, and establishing supportive settings for patients with BC. These strategies can help overcome sociocultural barriers, thereby improving mental health outcomes in South Asian patients with BC.

This study unveiled factors significantly associated with loneliness in middle-aged and older patients with BC. However, some limitations should be considered when interpreting our findings. First, the cross-sectional design hindered the establishment of causal relationships between loneliness and factors explored in this study. Second, the patients were recruited from only two hospitals in Indonesia, which might have limited the generalizability of our results. Finally, we did not consider potential risk factors such as personality traits and perceived social stigma.^50^^,^^51^ These factors may induce loneliness. Future studies should address these gaps and comprehensively explore the associations of loneliness with various factors.

## Conclusions

Our findings indicate that unemployment, social support, and frailty significantly influence loneliness in middle-aged and older patients with BC. Early assessment of employment status, social support, and frailty should be integrated into routine health-care services for these patients. Health-care providers, including clinical nurses and oncology case managers, should implement appropriate strategies, such as support groups, consultations, and alternative nonpharmacological interventions, to mitigate frailty and enhance social support, thereby preventing or managing loneliness in patients with BC. In addition, tailored occupational guidance should be provided to middle-aged and older patients with BC. In the future, longitudinal studies should be conducted for the early management or prevention of loneliness.

## Ethics statement

The study was approved by the Institutional Review Board of the Taipei Medical University (IRB No. N202206028) and by the Health Research Ethics Committee RSUP Dr. M. Djamil Padang Hospital (IRB No. LB.02.02/5.7/129/2022). Written informed consent was obtained from all participants.

## Funding

This study received no external funding.

## Declaration of competing interest

The authors declare that they have no known competing financial interests or personal relationships that could have appeared to influence the work reported in this paper.

## Data availability statement

The data that support the findings of this study are available from the corresponding author upon reasonable request.

## CRediT author contribution statement

**Hui-Chuan Huang, Kuan-Chia Lin**: Conceptualization, Methodology, Data curation, Formal analysis, Writing. **Leni Merdawati, Hui-Chen Lin**: Methodology, Writing – Original draft preparation. **Ya-Chin Wang, Kuan-Chia Lin**: Formal analysis, Writing – Revised draft preparation, Data curation. **Leni Merdawati, Hui-Chuan Huang:** Conceptualization, Methodology, Data collection, Writing – Original and Revised draft preparation. All authors had full access to all the data in the study, and the corresponding author had final responsibility for the decision to submit for publication. The corresponding author attests that all listed authors meet authorship criteria and that no others meeting the criteria have been omitted.

## Acknowledgments

We thank all breast cancer patients, project staff, the Central General Hospital, Dr. M. Damil Padang, and Andalas University Hospital, Padang, Indonesia.

## Declaration of Generative AI and AI-assisted technologies in the writing process

No AI tools/services were used during the preparation of this work.

## References

[1] Russell D., Peplau L.A., Cutrona C.E. (1980). The revised UCLA Loneliness Scale: concurrent and discriminant validity evidence. J Pers Soc Psychol.

[2] Cacioppo J.T., Cacioppo S. (2018). The growing problem of loneliness. Lancet.

[3] Coyle C.E., Dugan E. (2012). Social isolation, loneliness and health among older adults. J Aging Health.

[4] Liang Y., Hao G., Wu M., Hou L. (2022). Social isolation in adults with cancer: an evolutionary concept analysis. Front Psychol.

[5] Adams R.N., Mosher C.E., Winger J.G., Abonour R., Kroenke K. (2018). Cancer-related loneliness mediates the relationships between social constraints and symptoms among cancer patients. J Behav Med.

[6] Deckx L., van den Akker M., Buntinx F. (2014). Risk factors for loneliness in patients with cancer: a systematic literature review and meta-analysis. Eur J Oncol Nurs.

[7] Jaremka L.M., Fagundes C.P., Peng J. (2013). Loneliness promotes inflammation during acute stress. Psychol Sci.

[8] Kraav S.L., Awoyemi O., Junttila N. (2021). The effects of loneliness and social isolation on all-cause, injury, cancer, and CVD mortality in a cohort of middle-aged Finnish men. A prospective study. Aging Ment Health.

[9] Erzen E., Çikrikci Ö. (2018). The effect of loneliness on depression: a meta-analysis. Int J Soc Psychiatr.

[10] Marroquín B., Czamanski-Cohen J., Weihs K.L., Stanton A.L. (2016). Implicit loneliness, emotion regulation, and depressive symptoms in breast cancer survivors. J Behav Med.

[11] Jaremka L.M., Nadzan M.A. (2018). Threats to belonging among breast cancer survivors: consequences for mental and physical health. Current Breast Cancer Reports.

[12] Deckx L., van den Akker M., van Driel M. (2015). Loneliness in patients with cancer: the first year after cancer diagnosis. Psycho Oncol.

[13] Prates A.C.L., Freitas-Junior R., Prates M.F.O., Veloso MdF., Barros NdM. (2017). Influence of body image in women undergoing treatment for breast cancer. Rev Bras Ginecol Obstet.

[14] Thakur M., Sharma R., Mishra A.K., Singh K., Kar S.K. (2022). Psychological distress and body image disturbances after modified radical mastectomy among breast cancer survivors: a cross-sectional study from a tertiary care centre in North India. The Lancet Region Health-Southeast Asia.

[15] Tao J.J., Visvanathan K., Wolff A.C. (2015). Long term side effects of adjuvant chemotherapy in patients with early breast cancer. Breast.

[16] Tisnasari I.A.M.A.S., Nuraini T., Afiyanti Y. (2022). Stigma and discrimination against breast cancer survivors in Indonesia: an interpretive phenomenology study. Jurnal Ners.

[17] Ninnoni J.P., Agyemang S.O., Bennin L. (2023). Coping with loneliness and stigma associated with HIV in a resource-limited setting, making a case for mental health interventions; a sequential mixed methods study. BMC Psychiatr.

[18] Molina Y., Choi S.W., Cella D., Rao D. (2013). The stigma scale for chronic illnesses 8-item version (SSCI-8): development, validation and use across neurological conditions. Int J Behav Med.

[19] Polański J., Misiąg W., Chabowski M. (2022). Impact of loneliness on functioning in lung cancer patients. Int J Environ Res Public Health..

[20] Lemij A.A., de Glas N.A., Derks M.G.M. (2023). Mental health outcomes in older breast cancer survivors: five-year follow-up from the CLIMB study. Eur J Cancer.

[21] Su M., Yao N., Shang M. (2022). Frailty and its association with health-related quality of life among older cancer patients: an evidence-based study from China. Health Qual Life Outcomes.

[22] McElfresh J.J., Skiba M.B., Segrin C.G. (2021). Interventions for loneliness among adult cancer survivors: a systematic review and meta-analysis. J Psychosoc Oncol.

[23] Tabrizi F.M., Radfar M., Taei Z. (2016). Effects of supportive-expressive discussion groups on loneliness, hope and quality of life in breast cancer survivors: a randomized control trial. Psycho Oncol.

[24] Lenz E.R., Pugh L.C., Milligan R.A., Gift A., Suppe F. (1997). The middle-range theory of unpleasant symptoms: an update. Adv Nurs Sci.

[25] von Elm E., Altman D.G., Egger M., Pocock S.J., Gøtzsche P.C., Vandenbroucke J.P. (2007). Strengthening the Reporting of Observational Studies in Epidemiology (STROBE) statement: guidelines for reporting observational studies. BMJ.

[26] Solmi M., Veronese N., Galvano D. (2020). Factors associated with loneliness: an umbrella review of observational studies. J Affect Disord.

[27] Zhang X., Dong S. (Jul 2022). The relationships between social support and loneliness: a meta-analysis and review. Acta Psychol.

[28] Fauziyyah A., Ampuni S. (2018). Depression tendencies, social skills, and loneliness among college students in Yogyakarta. J Psikologi.

[29] McCorkle R., Young K. (1978). Development of a symptom distress scale. Cancer Nurs.

[30] McCorkle R., Cooley M., Shea J. (1998).

[31] Zimet G.D., Dahlem N.W., Zimet S.G., Farley G.K. (1988). The multidimensional scale of perceived social support. J Pers Assess.

[32] Winahyu K.M., Hemchayat M., Charoensuk S. (2015). The relationships between health status, perceived control of symptoms, caregiver burden, perceived social support and quality of life among family caregivers of patients with schizophrenia in Indonesia. J Prapokklao Hosp Clinic Med Edu Center.

[33] Schuurmans H., Steverink N., Lindenberg S., Frieswijk N., Slaets J.P. (2004). Old or frail: what tells us more?. J Gerontol Series A Biol Sci Med Sci.

[34] Steverink N. (2001). Measuring frailty: developing and testing the GFI (Groningen frailty indicator). Gerontol.

[35] Bielderman A., van der Schans C.P., van Lieshout M.-R.J. (2013). Multidimensional structure of the Groningen Frailty Indicator in community-dwelling older people. BMC Geriatr.

[36] Rico-Uribe L.A., Caballero F.F., Martín-María N., Cabello M., Ayuso-Mateos J.L., Miret M. (2018). Association of loneliness with all-cause mortality: a meta-analysis. PLoS One.

[37] Gerst-Emerson K., Jayawardhana J. (2015). Loneliness as a public health issue: the impact of loneliness on health care utilization among older adults. Am J Public Health.

[38] Morrish N., Medina-Lara A. (2021). Does unemployment lead to greater levels of loneliness? A systematic review. Soc Sci Med.

[39] Pohlan L. (2019). Unemployment and social exclusion. J Econ Behav Organ.

[40] Shaha M., Cox C.L., Talman K., Kelly D. (2008). Uncertainty in breast, prostate, and colorectal cancer: implications for supportive care. J Nurs Scholarsh.

[41] Kim Y.H., Choi K.S., Han K., Kim H.W. (2018). A psychological intervention programme for patients with breast cancer under chemotherapy and at a high risk of depression: a randomised clinical trial. J Clin Nurs.

[42] Menec V.H., Newall N.E., Mackenzie C.S., Shooshtari S., Nowicki S. (2020). Examining social isolation and loneliness in combination in relation to social support and psychological distress using Canadian Longitudinal Study of Aging (CLSA) data. PLoS One.

[43] Herrera-Badilla A., Navarrete-Reyes A.P., Amieva H., Avila-Funes J.A. (2015). Loneliness is associated with frailty in community-dwelling elderly adults. J Am Geriatr Soc.

[44] Strasser B., Steindorf K., Wiskemann J., Ulrich C.M. (2013). Impact of resistance training in cancer survivors: a meta-analysis. Med Sci Sports Exerc.

[45] Fox R.S., Ancoli-Israel S., Roesch S.C. (2020). Sleep disturbance and cancer-related fatigue symptom cluster in breast cancer patients undergoing chemotherapy. Support Care Cancer.

[46] Kołodziejczyk A., Pawłowski T. (2019). Negative body image in breast cancer patients. Adv Clin Exp Med.

[47] Zhu M., Zhang Y., He H., Chen L., Chen J., Zhang M. (2023). Social participation and acceptance of disability in young and middle-aged breast cancer patients after surgery: a 6-month follow-up study. Asia Pac J Oncol Nurs.

[48] Solikhah S., Matahari R., Utami F.P., Handayani L., Marwati T.A. (2020). Breast cancer stigma among Indonesian women: a case study of breast cancer patients. BMC Wom Health.

[49] Bedi M., Devins G.M. (Feb 2016). Cultural considerations for South Asian women with breast cancer. J Cancer Surviv..

[50] Gershfeld-Litvin A., Halabi S., Bellizzi K.M. (2022). Stigma related to breast cancer among women and men: the case of the Druze minority in Israel. J Health Psychol.

[51] Suwankhong D., Liamputtong P. (2016). Breast cancer treatment: experiences of changes and social stigma among Thai women in southern Thailand. Cancer Nurs.

